# Supplementary figures and images for: Novelty detection in early olfactory processing of the honey bee, Apis mellifera
Source: PLoS One. 2022 Mar 30;17(3):e0265009. doi: 10.1371/journal.pone.0265009 (PMC8967009; doi:10.1371/journal.pone.0265009)

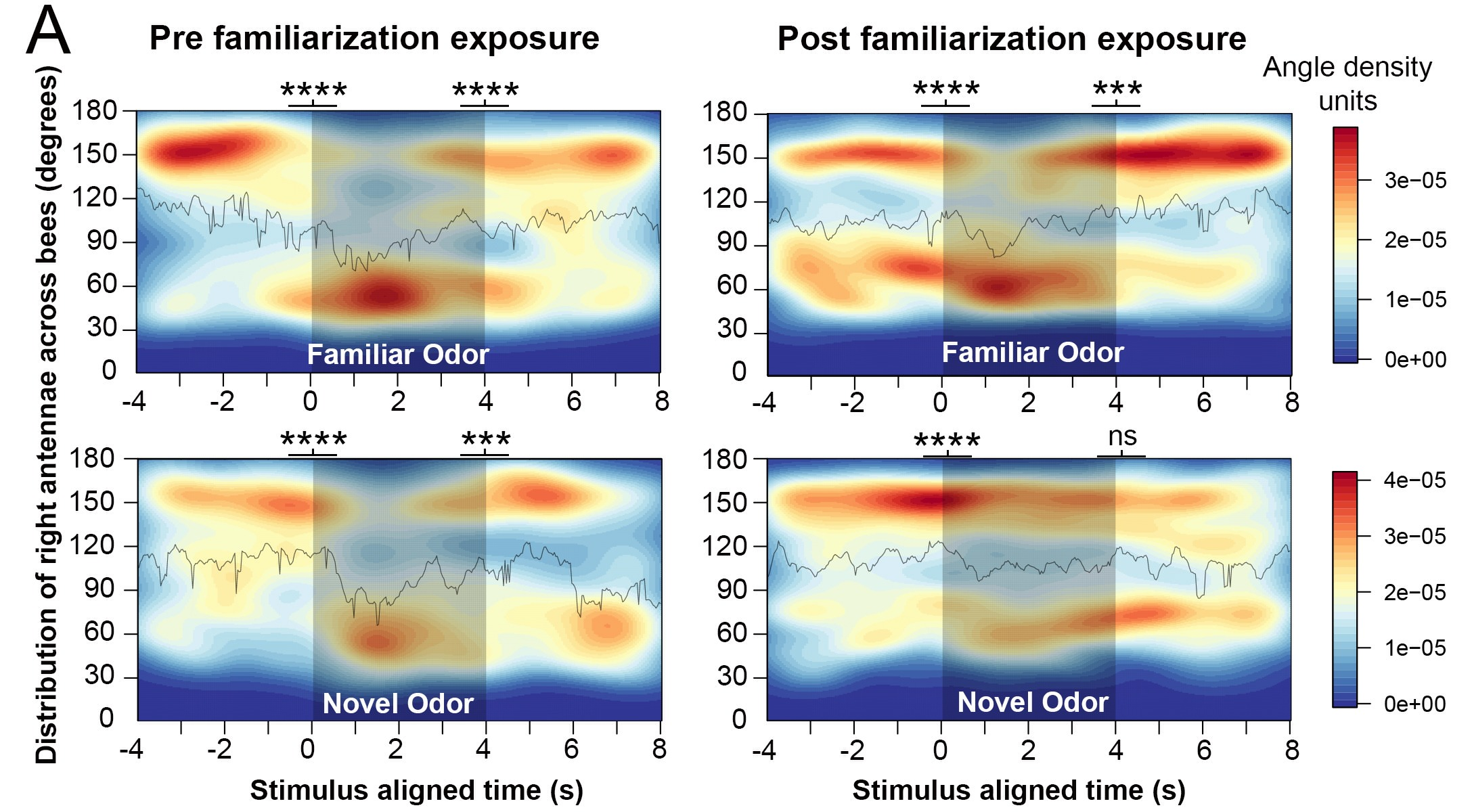

Supplement: S1 Fig — Angular positions are defined as in Fig 1. A. The average distribution densities of right antennae’s angular positions in response to odors before the familiarization process (left column plots) and 10 min after the familiarization process (right column plots) aligned by odor onset over 12 s Statistical analyses: Two-way ANOVA, difference measure~ time period * odor, df = 2, n = 24, Tukey HSD post-hoc comparison, asterisks indicate statistical significance, **** p<0.00001, ns indicates not significant. (TIF) [file pone.0265009.s001.tif]
